# Integrated CT Radiomics Features Could Enhance the Efficacy of ^18^F-FET PET for Non-Invasive Isocitrate Dehydrogenase Genotype Prediction in Adult Untreated Gliomas: A Retrospective Cohort Study

**DOI:** 10.3389/fonc.2021.772703

**Published:** 2021-11-19

**Authors:** Weiyan Zhou, Qi Huang, Jianbo Wen, Ming Li, Yuhua Zhu, Yan Liu, Yakang Dai, Yihui Guan, Zhirui Zhou, Tao Hua

**Affiliations:** ^1^ PET Center, Huashan Hospital, Fudan University, Shanghai, China; ^2^ Department of Radiology, Huashan Hospital, Fudan University, Shanghai, China; ^3^ Suzhou Institute of Biomedical Engineering and Technology, Chinese Academy of Sciences, Suzhou, China; ^4^ Jinan Guoke Medical Engineering Technology Development Co., LTD, Jinan, China; ^5^ Radiation Oncology Center, Huashan Hospital, Fudan University, Shanghai, China

**Keywords:** (18F)fluoroethyltyrosine, glioma, isocitrate dehydrogenase (IDH), radiomics, positron emission tomography (PET)

## Abstract

**Purpose:**

We aimed to investigate the predictive models based on O-[2-(^18^F)fluoroethyl]-l-tyrosine positron emission tomography/computed tomography (^18^F-FET PET/CT) radiomics features for the *isocitrate dehydrogenase (IDH)* genotype identification in adult gliomas.

**Methods:**

Fifty-eight consecutive pathologically confirmed adult glioma patients with pretreatment ^18^F-FET PET/CT were retrospectively enrolled. One hundred and five radiomics features were extracted for analysis in each modality. Three independent radiomics models (PET-Rad Model, CT-Rad Model and PET/CT-Rad Model) predicting *IDH* mutation status were generated using the least absolute shrinkage and selection operator (LASSO) regression analysis based on machine learning algorithms. All-subsets regression and cross validation were applied for the filter and calibration of the predictive radiomics models. Besides, semi-quantitative parameters including maximum, peak and mean tumor to background ratio (TBRmax, TBRpeak, TBRmean), standard deviation of glioma lesion standardized uptake value (SUV_SD_), metabolic tumor volume (MTV) and total lesion tracer uptake (TLU) were obtained and filtered for the simple model construction with clinical feature of brain midline involvement status. The area under the receiver operating characteristic curve (AUC) was applied for the evaluation of the predictive models.

**Results:**

The AUC of the simple predictive model consists of semi-quantitative parameter SUV_SD_ and dichotomized brain midline involvement status was 0.786 (95% CI 0.659-0.883). The AUC of PET-Rad Model building with three ^18^F-FET PET radiomics parameters was 0.812 (95% CI 0.688-0.902). The AUC of CT-Rad Model building with three co-registered CT radiomics parameters was 0.883 (95% CI 0.771-0.952). While the AUC of the combined ^18^F-FET PET/CT-Rad Model building with three CT and one PET radiomics features was 0.912 (95% CI 0.808-0.970). DeLong test results indicated the PET/CT-Rad Model outperformed the PET-Rad Model (*p* = 0.048) and simple predictive model (*p* = 0.034). Further combination of the PET/CT-Rad Model with the clinical feature of dichotomized tumor location status could slightly enhance the AUC to 0.917 (95% CI 0.814-0.973).

**Conclusion:**

The predictive model combining ^18^F-FET PET and integrated CT radiomics features could significantly enhance and well balance the non-invasive *IDH* genotype prediction in untreated gliomas, which is important in clinical decision making for personalized treatment.

## Introduction

Glioma is the most frequently-occurred primary malignant tumor in the brain. The 2016 WHO classification of the central nervous system tumor introduced a new integrated classification mode of glioma (1). *Isocitrate dehydrogenase* (*IDH*) mutation is considered to be an early event in the occurrence and development of glioma (2). *IDH* mutations were identified with a high percentage in low-grade gliomas and secondary glioblastoma multiforme (GBM) but with a much lower percentage in primary GBM (3).

*IDH* mutation altered the metabolism and microstructure of gliomas, thus affecting the biological characteristics and prognosis (4). Evidence supports that astrocytoma with *IDH* wildtype and other GBM-like molecular features has similar behavior as WHO grade IV glioma (5). At present, the identification of *IDH* mutation status was mainly based on surgical resection or biopsy specimens. However, additional surgical risks related to patient’s comorbidities, advanced age, deep-seated tumors are the barriers to accurately detect the *IDH* mutation status. Therefore, reliable methods which could non-invasively detect *IDH* mutation status are needed.

O-[2-(^18^F)fluoroethyl]-l-tyrosine (^18^F-FET) is an amino acid tracer of positron emission tomography (PET) imaging which could generally well outline the lesion out of the brain background, and could be applied as an effective complementary diagnostic modality besides magnetic resonance imaging (MRI) (6). The characteristic of ^18^F-FET PET imaging has led to wide clinical application (7–9), and gained the recommendation in all the phases of glioma management (10, 11).

Although these above-mentioned modalities could benefit the imaging diagnosis and evaluation, the intratumoral heterogeneity of glioma still challenge the precise imaging diagnosis and prediction. Our previous experience indicated conventional semi-quantitative parameters of static ^18^F-FET PET imaging could contribute to glioma non-invasive prediction in some degree (12). However, multiple dimensional and more detailed description of the imaging heterogeneity of glioma are crucial in improving the predictive efficacy. Radiomics technology could further extract imaging features among voxels for quantitative analysis based on the imaging heterogeneity, which could provide valuable information about tumor spatial and microenvironment (13). MRI radiomics has shown promising results in glioma imaging research (14). Limited research has shown the potential of amino acid PET imaging based radiomics analysis in glioma noninvasive grading, prognostication, pseudoprogression differentiation and molecular markers prediction such as *IDH* mutation (15–21). Computed tomography (CT) radiomics features, which has shown encouraging findings in lung, breast, gastrointestinal tract, pancreas and renal neoplasms, et al. (22–26), have rarely been applied for glioma radiomics analysis. Whether radiomics features of the amino acid tracer PET and co-registered CT images could contribute to glioma noninvasive prediction is of interest.

The purpose of this research was to investigate the efficacy of predictive radiomics models originating from PET modality, CT modality and combined PET/CT modalities generated with machine learning algorithms based on static ^18^F-FET PET/CT imaging in noninvasive *IDH* prediction, and to compare with the conventional semi-quantitative parameter analysis.

## Materials and Methods

### Patient Selection

We retrospectively analyzed the patients who received consecutive ^18^F-FET PET/CT brain imaging at the PET Center of Huashan Hospital, Fudan University from November 2017 to February 2019. A total of 58 untreated adult glioma patients were identified based on inclusion and exclusion criteria **Supplementary S1**. Baseline clinical and demographic characteristics including age, gender, tumor location, WHO grade, and *IDH* mutation status, were derived from medical file system of Huashan Hospital, Fudan University.

### ^18^F-FET PET/CT Imaging Protocol

All patients had fasted for at least 4 hours before imaging. 20 minutes after an intravenous bonus injection of ^18^F-FET (370 ± 30 MBq), a static PET scan was collected for 20 minutes with a Siemens Biograph 64 HD PET/CT (Siemens, Erlangen, Germany) in 3-dimensional (3D) mode for 50 patients in this cohort. 8 patients underwent dynamic ^18^F-FET PET scanning lasted longer than 40 minutes after the tracer injection; 20–40 minutes images were reconstructed for diagnosis and analysis. Attenuation correction was performed using a low-dose CT (tube current 150mAs, voltage 120kV, Acq. 64*0.6mm, convolution kernel H30s, slice thickness 5mm, interslice gap 1.5mm) before the emission scan. After acquisition, the PET images were reconstructed by filtered back projection algorithm with Gaussian filter and full-width-at-half-maximum at the center of the field of view of 3.5mm). The reconstructed brain PET image matrix size was 168*168 with voxel size of 2.04*2.04mm. And the reconstructed brain CT image matrix size was 512*512 with voxel size of 0.59*0.59 mm.

### Histological Evaluation and IDH Genotype Analysis

Histological specimens were obtained by surgical resection or stereotactic brain biopsy. H&E staining and immuno-histochemical analysis were performed by experienced neuropathologists according to the 2016 WHO classification. *IDH* genotype status was identified with an antibody to the *IDH1* (R132H) mutation by immunohistochemical staining. For 31 out of the 58 patients, *IDH* mutational status from the immuno-histochemical staining was further confirmed by sequencing.

### Conventional Threshold-Based ^18^F-FET PET Imaging Analysis

PET/CT imaging was first analyzed using a dedicated workstation (Siemens Syngo.*via*) to obtain semi-quantitative parameters in 3D volumes. Background mean standardized uptake value (SUV) was measured initially in a crescent-shape area, including both gray and white matter on the contralateral hemisphere as mentioned (27). The brain structural MRI of patients were reviewed initially to locate the tumor region. A predefined threshold of 1.6-times of the background mean SUV was applied for tumor volume of interest (VOI) delineation to derive lesion maximal SUV (SUVmax), peak SUV (SUVpeak), mean SUV (SUVmean), the standard deviation of lesion standardized uptake value (SUV_SD_), metabolic tumor volume (MTV) and total lesion tracer uptake (TLU) (28). Maximal tumor to background ratio (TBRmax), peak tumor to background ratio (TBRpeak), and mean tumor to background ratio (TBRmean) were calculated by the division of tumor VOI SUVmax, SUVpeak and SUVmean with background SUVmean. TLU was defined as the MTV multiplied by the tumor lesion SUVmean. For those multifocal glioma patients in our group, the specific surgical resected or biopsied lesion for pathological examination were included in our research to avoid bias.

Two experienced nuclear medicine physicians (WY Zhou: 5 years’ experience, T Hua: 10 years’ experience) performed lesion delineation and obtained two sets of semi-quantitative parameters separately. Brain midline involvement status were judged respectively by the two physicians after lesion delineation based on ^18^F-FET PET imaging. The brain midline structures included corpus callosum, cingulate gyrus, bilateral thalamus, third ventricle, brain stem and cerebellar vermis. The inter-observer agreement indices were evaluated for those obtained semi-quantitative parameters and glioma location status.

### Semi-Quantitative Parameters Filter and Simple Predictive Model Building

Univariate logistic regression was applied to filter the conventional semi-quantitative parameters for further model construction. The best-performance semi-quantitative parameter was combined with dichotomous brain midline involvement status for simple predictive model building.

### Segmentation and Radiomics Features Extraction

The original ^18^F-FET PET/CT data in digital imaging and communications in medicine (DICOM) format were converted into NIFTI format using SPM12 (Welcome Trust Centre for Neuroimaging, London, UK; http://www.fil.ion.ucl.ac.uk/spm).Then the tumor lesions were manually delineated on ^18^F-FET PET images using the ITK-SNAP software (http://www.itksnap.org/pmwiki/pmwiki.php) with the reference to patients’ structural MRI by the above-mentioned two physicians separately. If the divergence of a patient’s segmentation by the two physicians was less than 5%, the final segmentation of this specific patient was determined as the overlapping region of the two separate VOIs, and if the divergence was more than 5%, a senior nuclear medicine physician with over 25 years’ experience (YH Guan) made the final decision. After that the masks of those final tumor VOIs delineation on the ^18^F-FET PET images were applied to the co-registered CT images for the CT lesion delineation obtaining.

The registered ^18^F-FET PET/CT images and the VOIs were used to extract features. Prior to feature extraction, image standardization was implemented as follows: sitkBSpline interpolation resampling techniques were used to standardize the image scale in the slice, resulting in a pixel size of 2.5mm×2.5mm×2.5mm for PET and 1.5mm×1.5mm×1.5mm for CT, respectively. Two sets (105*2) of radiomics features from VOI lesions of the original PET and CT images were extracted with no further transformation or filtering using PyRadiomics (https://github.com/Radiomics/pyradiomics) (29), including 13 shape and size features, 18 first-order features and 74 texture features. The shape and size features included descriptors of the 3D size and shape of the VOI, which are independent from the gray level intensity distribution in the VOI. First-order features included the maximum, mean, and average absolute deviation of the gray-level intensity values in the VOIs. The texture features consisted of second-order features and are used to express heterogeneity in the tumor, from common matrices such as gray-level co-occurrence matrix (GLCM), gray-level size zone matrix (GLSZM), gray-level run length matrix (GLRLM), gray level dependence matrix (GLDM) and neighboring gray tone difference matrix (NGTDM). The feature extraction algorithms were standardized by referring to the Image Biomarker Standardization Initiative (IBSI) (30). Considering the relative low-resolution nature of PET images, radiomics feature extraction was conducted only on the original PET images with no further transformation or filtering. The details of extracted radiomics features were presented in **Supplementary S2**.

### Radiomics Features Filter and Predictive Model Establishment

Owing to the relatively small number of glioma patients in our cohort, less than five radiomics features were filtered for predictive model construction to minimize overfitting or selection bias in our radiomics models (13, 31). Least absolute shrinkage and selection operator (LASSO) and all-subsets regression were performed to filter the most significant radiomics features combinations for *IDH* genotyping. The LASSO algorithm adds a L1 regularization term to a least square algorithm to avoid overfitting, which is suitable for the regression of high dimensional data (32). Logistic regression analysis was then utilized to integrate the filtered features after further all-subsets regression selection. Calculated-score of three predictive models originating from radiomics features of PET, CT and their combined modalities were obtained by the linear fusion of the selected discriminating radiomics features weighted by their respective coefficients.

### Prediction Performance, Model Validation, and Calibration

The performance of the predictive models was evaluated by the receiver-operating characteristic (ROC) analysis and compared by the DeLong test. Additionally, the area under the ROC curve (AUC) with 95% confidence interval (CI), sensitivity, specificity, accuracy, positive predictive value and negative predictive value were calculated for each predictive model.

The four models for *IDH* genotype prediction were evaluated with k (k=3,5,10) fold cross-validation. The clinical application value of the predictive models was determined and compared through the decision curve analysis (DCA) by quantifying the net benefit to the patient under different threshold probabilities in the cohort (33).

### Statistical Analyses

Descriptive statistics were presented as mean ± standard deviation or median and range. Categorical variables were expressed as percentages. An independent sample t-test was used to compare two groups, while the chi-square test was applied to calculate *p* values for categorical variables. The Mann-Whitney rank sum test was used when variables were not normally distributed. Inter-observer agreements on ^18^F-FET PET metrics and dichotomized location results were assessed with interclass correlation coefficients (ICC) and Cohen’s kappa coefficient analysis respectively, defined as poor (less than 0.2), fair (0.21-0.4), moderate (0.41-0.6), good (0.61-0.8), and very good (0.8-1.0). Univariate and multivariate logistic regressions were used to identify the predictive factors for *IDH* mutation. The package “glmnet” was used to perform LASSO binary logistic regression analysis, and the “leaps” package to achieve all-subset regression algorithms, and the “bootstrap” package, to perform cross-validations by the R software, version 4.0.4 (http://www.r-project.org/). The ROC analyses and the DeLong test were performed by MedCalc for windows (version11.3.3.0, MedCalc software, Mariakierke, Belgium). All other statistical analysis was performed with Prism Software version 8.0 (GraphPad, San Diego. CA). In all analyses, p < 0.05 was considered to indicate statistical significance.

## Results

### Clinical Characteristics of Patients

**Figure 1:**
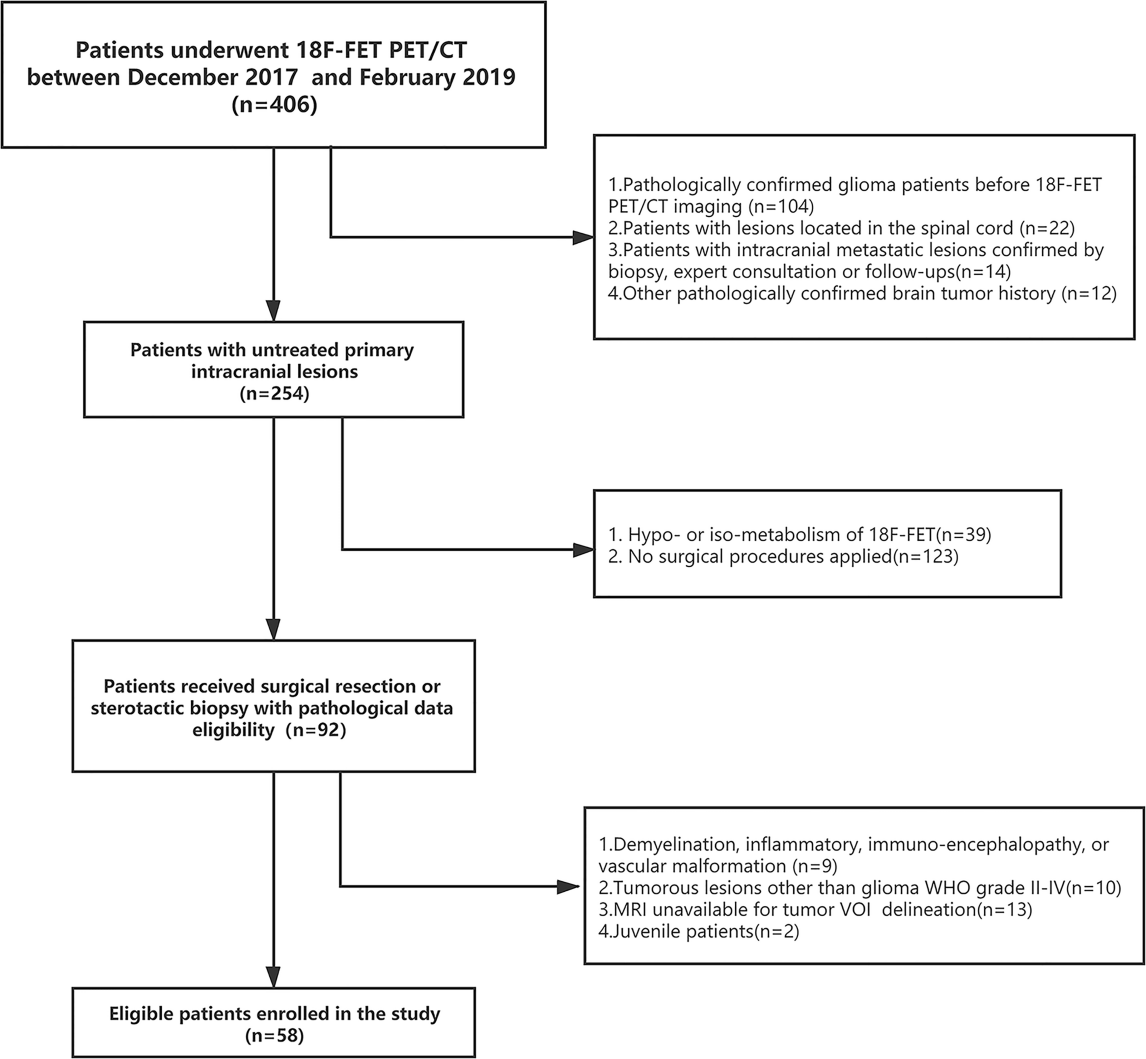
Flow chart showing the selection criteria of enrolled patients.

Demographic and clinical data of the 58 enrolled glioma patients were summarized in **Table 1**. The patient recruit process is presented in **Figure 1**. The time interval between PET imaging and subsequent tumor resection or biopsy was no more than 90 days for grade II or III patients and not exceeding 30 days for grade IV glioblastomas. A detailed chronologically enrolled patients’ clinical data was provided in **Supplementary S3**.

**Table 1:** Clinical features and ^18^F-FET PET semi-quantitative parameters based on *IDH* genotype.

Characteristics	*IDH*-mutant (n = 20)	*IDH*-wildtype (n = 38)	*P* Values
**Age** (median and range)	43 (24-68)	39.5 (17-70)	0.648
**Gender**			0.739
male	13 (65.00%)	23 (60.53%)	
female	7 (35.00%)	15 (39.47%)	
**WHO Grade**			***0.000***
II	18 (90.00%)	13 (34.21%)	
III	2 (10.00%)	12 (31.58%)	
IV	0 (00.00%)	13 (34.21%)	
**Operation Type**			***0.011***
total resection	13 (65.00%)	11 (28.95%)	
Subtotal resection	5 (25.00%)	10 (26.32%)	
Stereotactic biopsy	2 (10.00%)	17 (44.73%)	
**Midline Involvement**			*0.001*
yes	3 (15.00%)	24(63.16%)	
no	17(85.00%)	14 (36.84%)	
**^18^F-FET Metrics**			
TBRmax	2.5773 ± 0.8558 ^*^	3.1696 ± 1.2315 ^*^	0.069
TBRpeak	2.2451 ± 0.7071 ^*^	2.7435 ± 1.0729 ^*^	0.076
TBRmean	1.8488 ± 0.2912 ^*^	2.0331 ± 0.3592 ^*^	0.063
MTV	6.68 (2.47, 14.49)^#^	9.96 (4.69, 32.51)^#^	0.657
TLU	12.23 (2.82, 25.33)^#^	26.57 (11.97, 66.08)^#^	0.343
SUV_SD_	0.2145 ± 0.1577 ^*^	0.3782 ± 0.3158 ^*^	*0.045*

^18^F-FET,O-[2-(^18^F)-fluoroethyl]-l-tyrosine; TBR, tumor to background ratio; MTV, metabolic tumor volume; TLU, total lesion tracer uptake; SUV, standardized uptake value. ^*^Values refer to mean ± standard deviation. ^#^Values refer to median (interquartile range). P values were the results of univariate analysis of ecah characteristic except that bold ones indicated the results of chi-square tests.

### Inter-Reader Agreement for the Semi-Quantitative Parameters

The dichotomized brain midline involvement status of the interested tumor lesion yielded unified results for two separate readers, and the inter-observer kappa was satisfactory (κ=1.0, p<0.0001). The ICC also showed very good agreement for the ^18^F-FET PET volume-based semi-quantitative parameters (ICC>0.95, p<0.0001). Therefore, the results of reader one (WY Zhou) was applied for further analysis. The absolute values for all ^18^F-FET PET metrics based on different *IDH*-genotype were also provided in **Table 1**.

### Simple Predictive Model Building

Significant difference of SUV_SD_ (0.2145 ± 0.1577 *vs*. 0.3782 ± 0.3158, *p* = 0.034) and TLU [12.23(2.82-25.33) *vs*. 26.57(11.97-66.08), *p* = 0.046] could be observed in the two groups with different *IDH* genotype. While MTV of ^18^F-FET PET uptake was insignificant (*p*=0.239), the P values of TBRmax, TBRmean and TBRpeak were around the borderline (*p*=0.060, 0.053, 0.067, respectively). Further univariate logistic analysis results indicated that SUV_SD_ and the brain midline involvement status were significant independent predictors for *IDH* mutation. SUV_SD_ [≤0.23 *vs*. >0.23, odd ratio (OR): 3.208, 95%CI (1.013-10.163), *p* = 0.048] and brain midline involvement [no *vs*. yes incidence, OR: 9.714, 95%CI (2.412-39.125), *p* = 0.001] were associated with a higher of *IDH* mutation. Considering the collinearity of SUV_SD_ and TBRs (variance inflation factor threshold <5), SUV_SD_ and dichotomized tumor location status were selected to build the simple generalized linear model with reasonable *IDH* genotype predictive performance [AUC (95%CI) = 0.786 (0.659-0.883), sensitivity = 85.0%, specificity = 71.1%, accuracy = 75.9%]. The predictive scores of simple model for each patient were calculated using the following formula: 0.7836-2.1718*location (midline structure involved: 1, uninvolved: 0)-2.1462*SUV_SD._ *IDH*-mutants had higher calculated-scores than *IDH*-wildtypes in the simple model (-0.0025 ± 0.9398 *vs*. -1.3997 ± 1.2489, p < 0.001).

### Radiomics Models Performance and Calculated-Score Analyses

**Figure 2:**
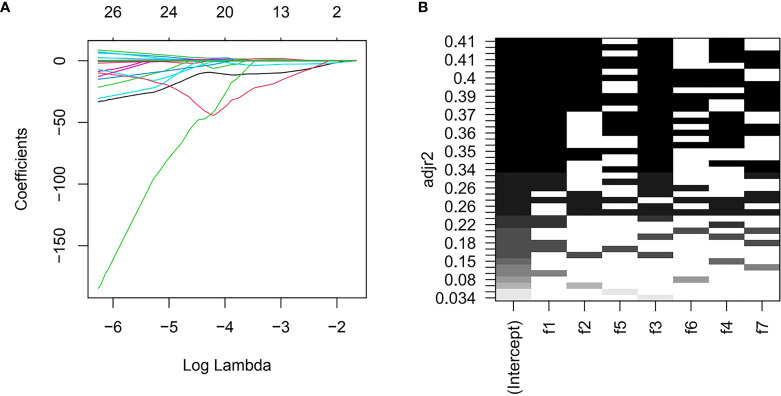
Features selection for PET/CT-Rad Model. **(A)** LASSO regression coefficient of the selected features could change with log (lambda). **(B)** further all-subsets regression based on seven features filtered by LASSO. adjr2, adjusted R square; f1, CT_glcm_InverseVariance; f2, CT_glrlm_LowGrayLevelRunEmphasis; f3, CT_gldm_DependenceVariance; f4, PET_glcm_JointEnergy; f5, CT_glszm_LargeAreaEmphasis; f6, PET_firstorder_Maximum; f7, PET_gldm_LargeDependenceLowGrayLevelEmphasis.

**Figure 3:**
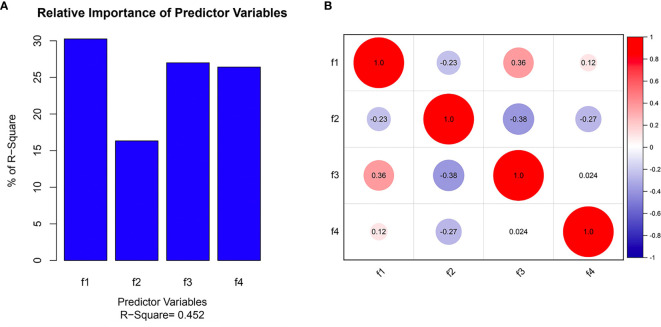
Selected features in the PET/CT-Rad model predicting *IDH* mutation. **(A)** Feature importance of selected features; **(B)** correlation heatmap of selected features. f1, CT_glcm_InverseVariance; f2, CT_glrlm_LowGrayLevelRunEmphasis; f3, CT_gldm_DependenceVariance; f4, PET_glcm_JointEnergy.


Three radiomics predictive models were developed by machine-learning based algorithms. Three PET radiomics features were selected for the building of the PET-Rad Model. Three CT radiomics features were selected for the building of the CT-Rad Model. Three CT radiomics features and one PET radiomics feature were filtered for the building of the PET/CT-Rad Model (shown in **Figure 2**). The relative importance analysis of the PET/CT-Rad Model indicated one of the three CT radiomics features (CT_glcm_InverseVariance) had the most important weight with 30.25% and the PET radiomics feature (PET_glcm_JointEnergy) had the third weight out of the four features (26.41%) in the PET/CT-Rad Model (shown in **Figure 3**). The calculated-scores of the three radiomics models for each patient were obtained using related formulas and shown as follows:


$$\begin{array}{l} Calculated-score\;(CT-Rad\;Model)=-3.55667-0.03504\\ {}*CT\_Maximum2DDiameterSlice-41.59021\\ {}*CT\_glcm\_InverseVariance+0.18756\\ {}*CT\_gldm\_DependenceVariance \end{array}$$

$$\begin{array}{l} Calculated-score\text{ (}PET-Rad\;Model)=101.7289-0.3486\\ {}*PET\_firstorder\_Maximum+0.8448\\ {}*PET\_gldm\_DependenceEntropy-75.1484\\ {}*PET\_gldm\_LargeDependenceLowGrayLevelEmphasis \end{array}$$

$$\begin{array}{l} Calculated-score\text{ (}PET/CT-Rad\;Model)=-5.5892-30.1241\\ {}*CT\_glcm\_InverseVariance+2.7319\\ {}*CT\_glrlm\_LowGrayLevelRunEmphasis+0.1643\\ {}*CT\_gldm\_DependenceVariance-7.3066*PET\_glcm\_JointEnergy \end{array}$$

**Figure 4:**
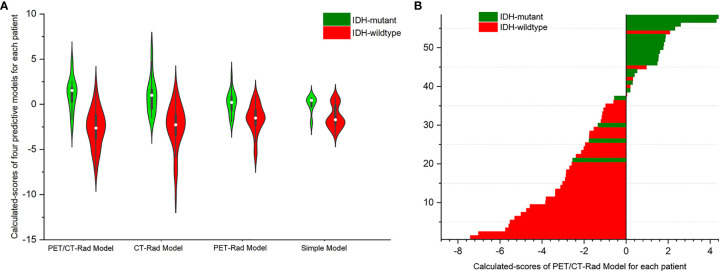
**(A)** Violin plot of three radiomics models indicated different *IDH* genotypes has different calculated-scores. The white dot represents the median. The black rectangle is the range from the lower quartile to the upper quartile. The black line running up and down through the violin diagram represents the range from the minimal non-outlier value to the maximal non-outlier value. **(B)** Calculated-scores of the PET/CT-Rad Model for each patient in the glioma patient cohort.

Results showed *IDH*-mutants had higher calculated-scores than *IDH*-wildtypes in the PET-Rad, CT-Rad and combined PET/CT-Rad models (1.1262 ± 1.8048 *vs*. -2.6648 ± 2.1637, *p* < 0.001; 1.0008 ± .2.0396 *vs*. -2.5214 ± 2.5610, *p* < 0.001; 0.1536 ± 1.2660*vs*. -1.6186 ± 1.6268, *p* < 0.001; respectively) (**Figures 4A**, **B**).

### Distinction of IDH-Mutants From IDH-Wildtypes Using Different Predictive Models

**Figure 5:**
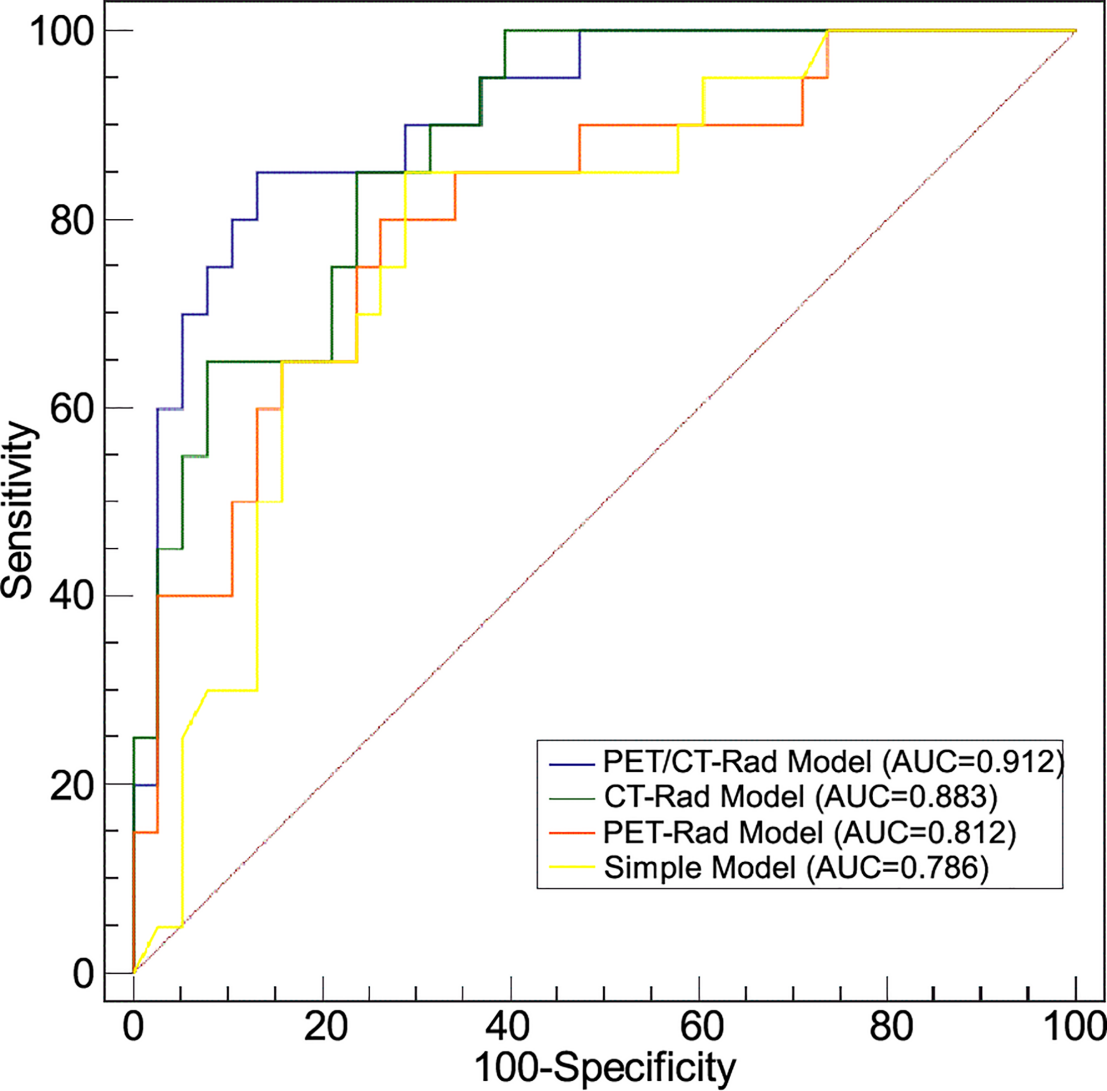
Receiver operating characteristic analyses of four predictive models for *IDH* genotype differentiation.

ROC analysis results indicated the AUC of the PET-Rad Model was 0.812 (95% CI 0.688-0.902), with a sensitivity of 80.0%, a specificity of 73.7% and an accuracy of 75.9%. The AUC of the CT-Rad Model was 0.883 (95% CI 0.771-0.952), with a sensitivity of 85.0%, a specificity of 76.3% and an accuracy of 79.3%. The PET/CT-Rad Model achieved the highest AUC (0.912, 95%CI 0.808-0.970), with a sensitivity of 85.0%, a specificity of 86.8% and an accuracy of 86.2% (Details were provided in **Table 2**). DeLong test results indicated that PET/CT-Rad Model outperformed the PET-Rad Model and the simple model in *IDH* genotype prediction (p = 0.048 and p = 0.034, respectively) (**Figure 5**). No statistically significant difference could be observed among the PET-Rad Model, CT-Rad Model and simple model.

**Table 2:** The performance of simple and three radiomics models.

	Simple Model	PET-Rad Model	CT-Rad Model	PET/CT-Rad Model
Included features	Location	PET_firstorder_Maximum	CT_glcm_InverseVariance	CT_glcm_InverseVariance
	SUV_SD_	PET_gldm_DependenceEntropy	CT_Maximum2DDiameterSlice	CT_glrlm_LowGrayLevelRunEmphasis
		PET_gldm_LargeDependenceLowGrayLevelEmphasis	CT_gldm_DependenceVariance	CT_gldm_DependenceVariance
				PET_glcm_JointEnergy
Accuracy	75.9%	75.9%	79.3%	86.2%
Sensitivity	85.0%	80.0%	85.0%	85.0%
Specificity	71.1%	73.7%	76.3%	86.8%
PPV	60.7%	61.5%	65.4%	77.3%
NPV	90.0%	87.5%	90.6%	91.7%
AUC(95%CI)	0.786(0.659-0.883)	0.812(0.688-0.902)	0.883(0.771-0.952)	0.912(0.808-0.970)
AIC	64.29	64.43	53.18	50.18
Original R^2^	0.2605	0.2788	0.4206	0.5175
10-Fold CV R^2^	0.2153	0.1860	0.3043	0.3901
5-Fold CV R^2^	0.2207	0.1479	0.3192	0.3968
3-Fold CV R^2^	0.2098	0.1910	0.3009	0.2696

PPV, positive predictive values; NPV, negative predctive values; AUC, area under the curve; CI, confidence interval; AIC, Akakike Information Criteria; CV, cross validation; Location, represents brain midline involvement status; Rad, radiomics; SUV, standardized uptake value.

**Figure 6:**
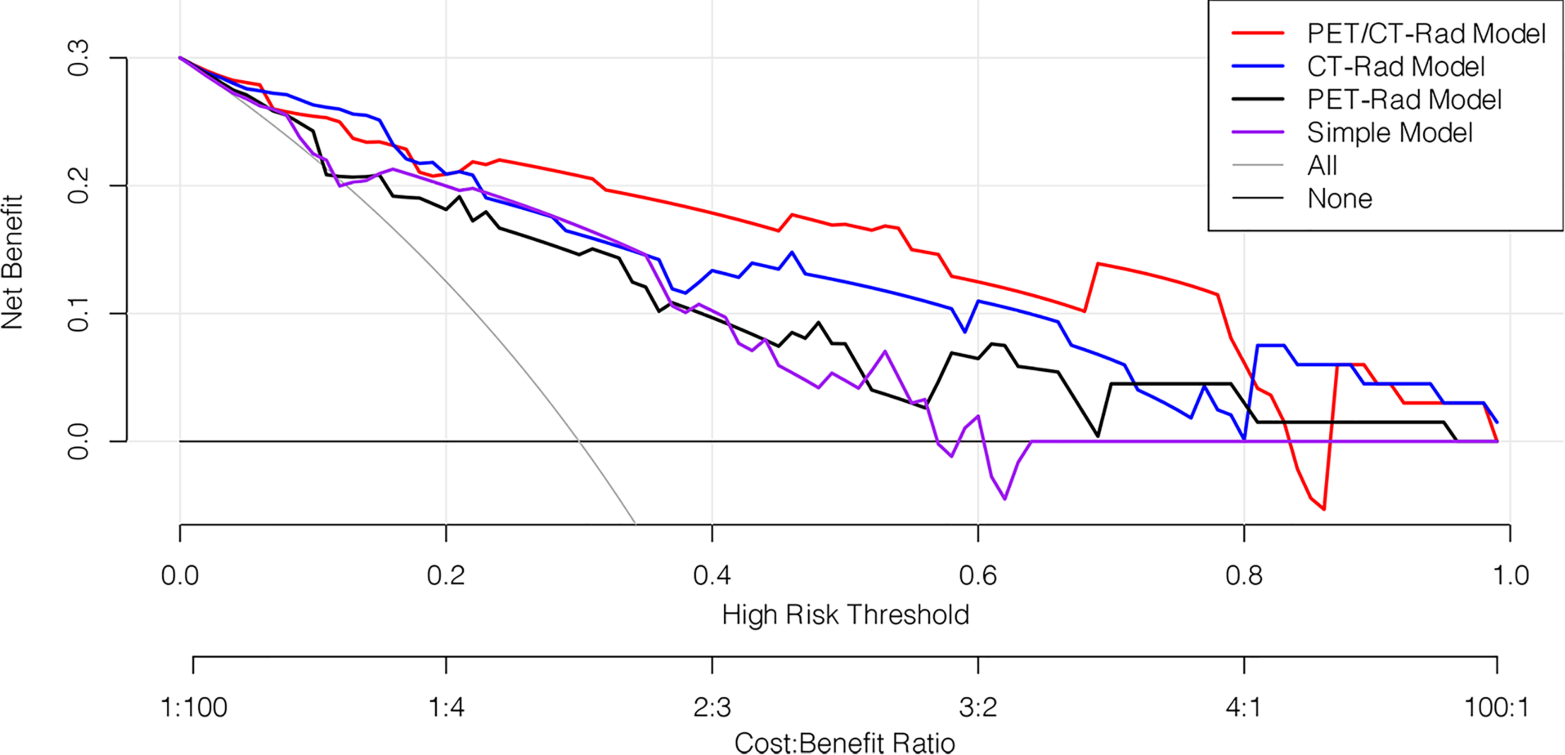
Decision curve analysis of four predictive models. The horizontal axis represented the threshold probability where the expected benefit of treatment as *IDH* mutation was equal to the expected benefit of avoiding treatment as *IDH* wildtype. The vertical axis represented the net benefit for the treatment which considered the benefit of true positive and loss of false positive. The net benefit of all these four models is further compared with the default strategies, which we treat all patients as *IDH* mutation (grey line) or as *IDH* wildtype (black horizontal line). Results indicated PET/CT-Rad model have the most satisfactory clinical value.

**Figure 7:**
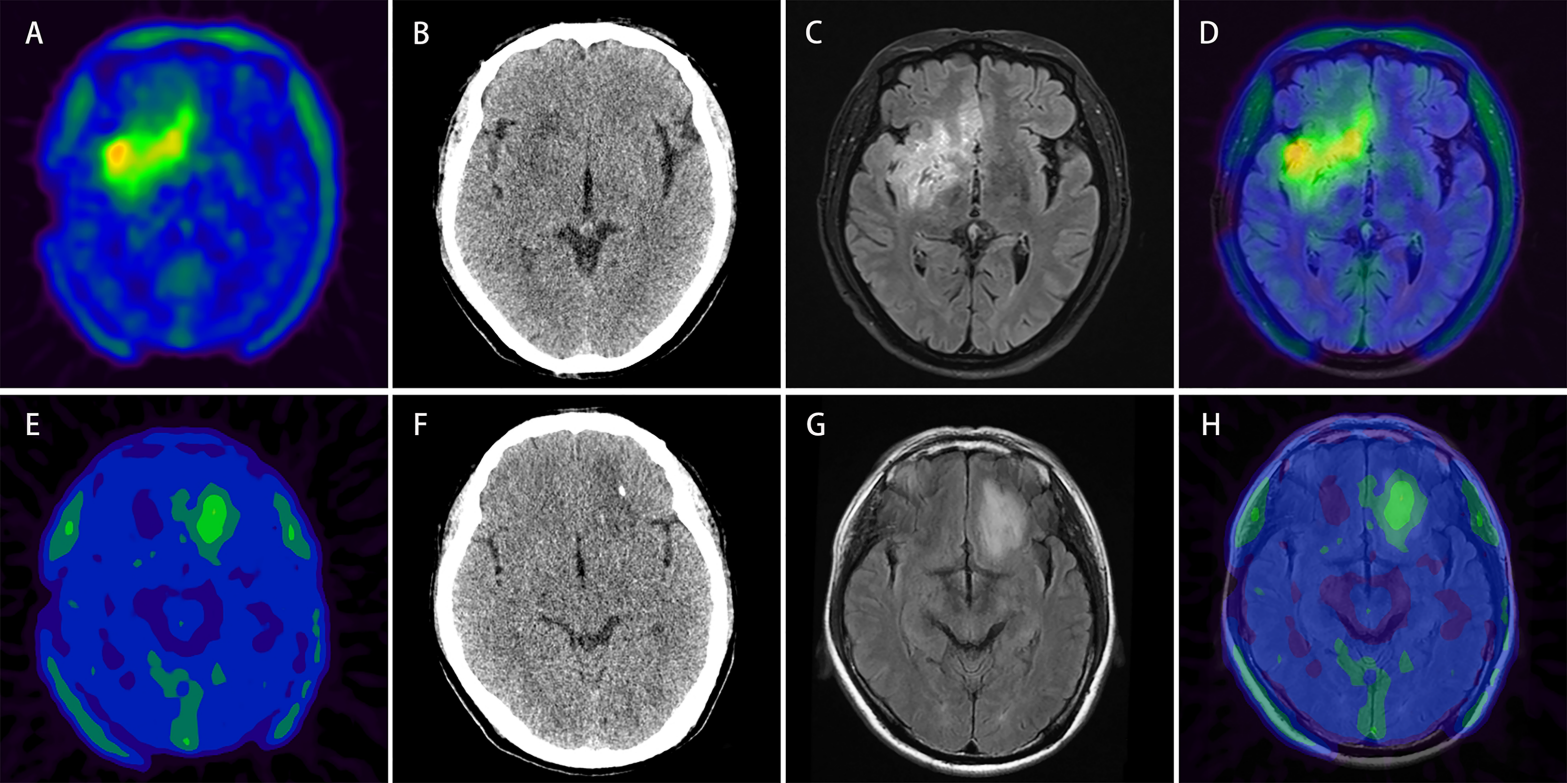
Upper row: example of a patient (patient No. 2) with ^18^F-FET lesion in the right frontotemporal-insular lobe infiltrating the brain midline structure **(A)** relatively more intratumoral heterogeneous imaging with a SUV_SD_ of 0.32 (> 0.23) and calculated-scores for the four predictive models (simple model, PET-Rad model, CT-Rad model and PET/CT-Rad model) were -2.07, -1.82, -1.19, and -3.02. **(B)** Corresponding low dose CT image of the patient **(C, D)** T2 FLAIR MRI and PET/MR fused image; histopathological analysis revealed a WHO grade II diffuse astrocytoma, *IDH*-wildtype. Lower row: example of a patient (patient No. 28) with ^18^F-FET lesion in the left frontal lobe without brain midline structure involvement **(E)** relatively less intratumoral heterogeneous imaging with a SUV_SD_ of 0.17 (< 0.23) and calculated-scores for the 4 predictive models were 0.42, 0.18, 1.80, and 0.53 **(F)** Corresponding low dose CT image of the patient **(G, H)** T2 FLAIR MRI and PET/MR fused image; histopathological analysis revealed a WHO grade II diffuse astrocytoma, *IDH*-mutant.

The decision curve analysis results revealed that the PET/CT-Rad Model was the most satisfactory predictive model in differentiating *IDH* mutation status in these four models (shown in **Figure 6**). Representative glioma patients of different *IDH* genotypes were provided in **Figure 7**.

### Predictive Efficacy of the Combination of PET/CT-Rad Model With Dichotomized Brain Midline Involvement Status

We further explored the combination of the dichotomized tumor location status and PET/CT-Rad Model for *IDH* mutation status prediction, and results indicated the predictive efficacy slightly enhanced in terms of AUC and accuracy (from 0.912 to 0.917 and 86.2% to 87.9%, respectively), although the enhancement was statistically insignificant.

## Discussion

Intratumoral heterogeneity is a significant feature of malignant tumors. Radiomics techniques have provided the quantification possibility to further investigate the imaging heterogeneity in internal space geometry, texture, gray distribution and shape (34). In our research we investigated the feasibility for PET, CT and PET/CT radiomics models for *IDH* mutation status prediction using LASSO regression based on static ^18^F-FET PET/CT imaging in glioma patients. After the combination of CT radiomics features, the PET/CT-Rad Model significantly outperformed ^18^F-FET PET-Rad Model and simple model in noninvasive *IDH* status prediction. Our investigation results could contribute to the further understanding of the relationship between the intratumoral heterogeneity (e.g., tumor cellularity, chaotic vascularization or necrosis, and tumor-related macrophage infiltration) with CT radiomics features beyond visual perception and amino acid tracer metabolism patterns in glioma lesions.

Reconstruction algorithms are crucial in PET/CT imaging analysis. The semi-quantitative parameters and radiomics features of ^18^F-FET PET imaging are affected by the reconstruction algorithms. Iterative and filtered back projection reconstructions are the two recommended methods in amino acid tracer PET imaging (6). Owing to the relatively low resolution of PET imaging, the efficacy of PET radiomics features needs to be enhanced and stabilized with the combination of structural radiomics features. One major obstacle for CT radiomics application in glioma was the VOI delineation process, which amino acid tracer PET imaging could well complement. With the duplication of the ^18^F-FET PET lesion VOI delineation to the co-registered CT imaging, we could evaluate the efficacy of CT radiomics features. The CT radiomics predictive model comprises both shape and textural features from the glcm, and gldm matrixes. An intuitive interpretation of the signature might be that relatively homogeneous tumors with less aggressive growth pattern is in concordance with relatively favorable *IDH* genotype. Results in our research showed that after the combination of the co-registered CT imaging radiomics features, the predictive accuracy, sensitivity, specificity and AUC of the ^18^F-FET PET/CT radiomics model could be enhanced and stabilized in noninvasive *IDH* genotype prediction.

Different weighted images in MRI could benefit significantly in brain imaging. A recent study by Bangalore et al. evaluated the usefulness of T2-weighted MRI-based deep learning method for the determination of *IDH* mutation status (35). The results are inspiring since T2-weighted MRI is widely available and routinely performed in the assessment of gliomas. Owing to the incomplete structural MRI DICOM data of our patient cohort, we investigated the radiomics features of integrated CT images in our research. Results showed CT radiomics features have promising potentials in the *IDH* genotyping of glioma patients.

SUV_SD_ is a semi-quantitative parameter based on static ^18^F-FET PET images which could reflect the metabolic heterogeneity in some degree. We have confirmed the efficacy of this semi-quantitative parameter in ^11^C-MET and ^18^F-FET PET imaging in glioma patients for *IDH* mutation status prediction (12, 36). However, cautions should be noticed in interpreting this specific semi-quantitative parameter because the reproducible lesion delineation procedures are the prerequisite for the application of SUV_SD._ In this patient cohort, the most frequently used semi-quantitative parameters including TBRmax and TBRmean were found to have borderline significance in differentiating *IDH* mutation status. With the enrollment number increases, further investigation could be applied to observe the efficacy of these classical semi-quantitative parameters.

Although the radiomics signatures and simple model have shown efficacy in *IDH* mutation status prediction, we must know that all these imaging analyses were based on robust and repetitive lesion delineation procedures. Experienced manual segmentation of glioma with the reference of structural MRI before radiomics features extraction, 1.6 times of contralateral brain background SUVmean with correct and reproducible tumor delineation for the acquirement of semi-quantitative parameters are crucial for this study. Additionally, the methodologies we used were a 3D classification approach without the concern of data leakage or the risk of introducing bias, compared to those 2D slice-wise-built models (37). With the application of radiomics techniques, abundant imaging features could be extracted for analysis. In this process caution should be made to avoid overfitting during the radiomics analysis, which could lead to bias in result interpretations. With the consideration of the numbers of our patient cohort and radiomics features, radiomics models in our research each consisting of less than five features would be acceptable to control possible overfitting. Cross-validations have been applied to evaluate the generalization of these predictive models owing to the lack of external validation.

Dynamic ^18^F-FET imaging could provide additional valuable information besides regular static parameters including lesion time activity curve pattern, time to peak, and slope. These parameters could also enhance the performance of static imaging parameters for *IDH* mutation detection (38–40). Moreover, the combination of dynamic parameters of ^18^F-FET imaging and radiomics features could benefit the non-invasive *IDH* genotype prediction (19, 41). In our research, dichotomized tumor location status could slightly enhance the efficacy of PET/CT-Rad Model (AUC from 0.912 to 0.917 and accuracy 86.2% to 87.9%, respectively). With the increasing enrollment number of glioma patients, we look forward to further investigate the results of radiomics features with the addition of the ^18^F-FET tracer kinetics and possible clinical features.

Although the amino acid tracer PET imaging has been recommended for the glioma diagnosis and evaluation, evidence showed a small portion of glioma patients could have iso- or hypo-FET uptake than the brain background (42). Limited to the method outling the tumor VOI, our filtered radiomics model is not generally applicable for the possible glioma patients with the iso- or hypo-FET uptake. With the update of the follow-up information in our facility, methodological optimization could further facilitate the better understanding of the ^18^F-FET PET/CT imaging heterogeneity in glioma patients.

There are limitations in present research. The retrospective nature of the study, the relatively low patient number, incomplete molecular biomarkers results (43), and lack of external validation limit the strength of the results. Radiomics analysis with PET/CT is substantially influenced by scanning protocol, image acquisition parameters and reconstruction algorithms (22). Harmonization and standardization for the radiomics methodology is essential for results reproductivity. Finally, the percentage of *IDH* mutation in our patient cohort was not well-balanced, the possible reason was that some imaging-typical glioma patients will not be recommended for amino acid tracer PET imaging owing to social-economic reasons. Thus, the general application of the radiomics model needs to be verified in an independent validation cohort with larger sample size and more comprehensive molecular biomarker information in future studies.

## Conclusions

In conclusion, we established three predictive radiomics models and one simple model using static ^18^F-FET PET/CT images for the non-invasive identification of *IDH* genotype in adult untreated glioma patients. The combination of co-registered CT and ^18^F-FET PET radiomics features could significantly enhance and well balance the *IDH* genotype prediction, which is crucial in treatment planning and prognostic evaluation in glioma patients.

## Data Availability Statement

The original contributions presented in the study are included in the article/**Supplementary Material**. Further inquiries can be directed to the corresponding authors.

## Ethics Statement

The studies involving human participants were reviewed and approved by the Ethics Committee of HuaShan Hospital Fudan University. Written informed consent to participate in this study was provided by the participants’ legal guardian/next of kin.

## Author Contributions

All authors listed have made a substantial, direct and intellectual contribution to the work, and approved it for publication.

## Funding

This research was partially supported by Science and Technology Commission of Shanghai Municipality (grant Nos.18411952100 and 2018SHZDZX01), and ZJ Lab; the Jinan Innovation Team (grant No. 2018GXRC017) and the Quancheng 5150 Project.

## Conflict of Interest

Authors YL and YD were employed by Jinan Guoke Medical Engineering Technology Development Co., LTD.

The remaining authors declare that the research was conducted in the absence of any commercial or financial relationships that could be construed as a potential conflict of interest.

## Publisher’s Note

All claims expressed in this article are solely those of the authors and do not necessarily represent those of their affiliated organizations, or those of the publisher, the editors and the reviewers. Any product that may be evaluated in this article, or claim that may be made by its manufacturer, is not guaranteed or endorsed by the publisher.

## References

[1] Louis DN Perry A Reifenberger G von Deimling A Figarella-Branger D Cavenee WK. The 2016 World Health Organization Classification of Tumors of the Central Nervous System: A Summary. Acta Neuropathol (2016) 131:803–20. doi: 10.1007/s00401-016-1545-1 27157931

[2] Turkalp Z Karamchandani J Das S. IDH Mutation in Glioma: New Insights and Promises for the Future. JAMA Neurol (2014) 71:1319–25. doi: 10.1001/jamaneurol.2014.1205 25155243

[3] Sun H Yin L Li S Han S Song G Liu N. Prognostic Significance of IDH Mutation in Adult Low-Grade Gliomas: A Meta-Analysis. J Neurooncol (2013) 113:277–84. doi: 10.1007/s11060-013-1107-5 23504258

[4] Wick W Hartmann C Engel C Stoffels M Felsberg J Stockhammer F. NOA-04 Randomized Phase III Trial of Sequential Radiochemotherapy of Anaplastic Glioma With Procarbazine, Lomustine, and Vincristine or Temozolomide. J Clin Oncol (2009) 27:5874–80. doi: 10.1200/JCO.2009.23.6497 19901110

[5] Brat DJ Aldape K Colman H Holland EC Louis DN Jenkins RB. cIMPACT-NOW Update 3: Recommended Diagnostic Criteria for “Diffuse Astrocytic Glioma, IDH-Wildtype, With Molecular Features of Glioblastoma, WHO Grade IV”. Acta Neuropathol (2018) 136:805–10. doi: 10.1007/s00401-018-1913-0 PMC6204285 30259105

[6] Law I Albert NL Arbizu J Boellaard R Drzezga A Galldiks N. Joint EANM/EANO/RANO Practice Guidelines/SNMMI Procedure Standards for Imaging of Gliomas Using PET With Radiolabelled Amino Acids and [(18)F]FDG: Version 1.0. Eur J Nucl Med Mol Imaging (2019) 46:540–57. doi: 10.1007/s00259-018-4207-9 PMC6351513 30519867

[7] Ogawa T Kawai N Miyake K Shinomiya A Yamamoto Y Nishiyama Y. Diagnostic Value of PET/CT With (11)C-Methionine (MET) and (18)F-Fluorothymidine (FLT) in Newly Diagnosed Glioma Based on the 2016 WHO Classification. EJNMMI Res (2020) 10:44. doi: 10.1186/s13550-020-00633-1 32382870 PMC7205963

[8] Takei H Shinoda J Ikuta S Maruyama T Muragaki Y Kawasaki T. Usefulness of Positron Emission Tomography for Differentiating Gliomas According to the 2016 World Health Organization Classification of Tumors of the Central Nervous System. J Neurosurg (2020) 133:1010–9. doi: 10.3171/2019.5.JNS19780 31419796

[9] Kebir S Weber M Lazaridis L Deuschl C Schmidt T Monninghoff C. Hybrid 11c-MET PET/MRI Combined With “Machine Learning” in Glioma Diagnosis According to the Revised Glioma WHO Classification 2016. Clin Nucl Med (2019) 44:214–20. doi: 10.1097/RLU.0000000000002398 30516675

[10] Bashir A Mathilde Jacobsen S Molby Henriksen O Broholm H Urup T Grunnet K. Recurrent Glioblastoma *Versus* Late Posttreatment Changes: Diagnostic Accuracy of O-(2-[18F]Fluoroethyl)-L-Tyrosine Positron Emission Tomography (18F-FET PET). Neuro Oncol (2019) 21:1595–606. doi: 10.1093/neuonc/noz166 PMC6917428 31618420

[11] Song S Cheng Y Ma J Wang L Dong C Wei Y. Simultaneous FET-PET and Contrast-Enhanced MRI Based on Hybrid PET/MR Improves Delineation of Tumor Spatial Biodistribution in Gliomas: A Biopsy Validation Study. Eur J Nucl Med Mol Imaging (2020) 47:1458–67. doi: 10.1007/s00259-019-04656-2 PMC7188715 31919633

[12] Hua T Zhou W Zhou Z Guan Y Li M. Heterogeneous Parameters Based on (18)F-FET PET Imaging can non-Invasively Predict Tumor Grade and Isocitrate Dehydrogenase Gene 1 Mutation in Untreated Gliomas. Quant Imaging Med Surg (2021) 11:317–27. doi: 10.21037/qims-20-723 PMC7719943 33392031

[13] Lambin P Leijenaar RTH Deist TM Peerlings J de Jong EEC van Timmeren J. Radiomics: The Bridge Between Medical Imaging and Personalized Medicine. Nat Rev Clin Oncol (2017) 14:749–62. doi: 10.1038/nrclinonc.2017.141 28975929

[14] Lohmann P Kocher M Steger J Galldiks N. Radiomics Derived From Amino-Acid PET and Conventional MRI in Patients With High-Grade Gliomas. Q J Nucl Med Mol Imaging (2018) 62:272–80. doi: 10.23736/S1824-4785.18.03095-9 29869488

[15] Papp L Potsch N Grahovac M Schmidbauer V Woehrer A Preusser M. Glioma Survival Prediction With Combined Analysis of *In Vivo* (11)C-MET PET Features, *Ex Vivo* Features, and Patient Features by Supervised Machine Learning. J Nucl Med (2018) 59:892–9. doi: 10.2967/jnumed.117.202267 29175980

[16] Pyka T Gempt J Hiob D Ringel F Schlegel J Bette S. Textural Analysis of Pre-Therapeutic [18F]-FET-PET and Its Correlation With Tumor Grade and Patient Survival in High-Grade Gliomas. Eur J Nucl Med Mol Imaging (2016) 43:133–41. doi: 10.1007/s00259-015-3140-4 26219871

[17] Kebir S Khurshid Z Gaertner FC Essler M Hattingen E Fimmers R. Unsupervised Consensus Cluster Analysis of [18F]-Fluoroethyl-L-Tyrosine Positron Emission Tomography Identified Textural Features for the Diagnosis of Pseudoprogression in High-Grade Glioma. Oncotarget (2017) 8:8294–304. doi: 10.18632/oncotarget.14166 PMC5352401 28030820

[18] Zhao K Yu P Xue Z Liu J Yao A Zhao Y. (11)C-Methionine Integrated PET/MRI-Based Texture Analysis Features May Have a Potential Ability to Distinguish Oligodendroglioma (IDH-Mutant and 1p/19q-Codeleted) From Varied Gliomas. Acad Radiol (2020) 27:e159–67. doi: 10.1016/j.acra.2019.09.013 31607471

[19] Lohmann P Lerche C Bauer EK Steger J Stoffels G Blau T. Predicting IDH Genotype in Gliomas Using FET PET Radiomics. Sci Rep (2018) 8:13328. doi: 10.1038/s41598-018-31806-7 30190592 PMC6127131

[20] Lohmann P Kocher M Ceccon G Bauer EK Stoffels G Viswanathan S. Combined FET PET/MRI Radiomics Differentiates Radiation Injury From Recurrent Brain Metastasis. NeuroImage Clin (2018) 20:537–42. doi: 10.1016/j.nicl.2018.08.024 PMC6118093 30175040

[21] Lohmann P Stoffels G Ceccon G Rapp M Sabel M Filss CP. Radiation Injury *vs*. Recurrent Brain Metastasis: Combining Textural Feature Radiomics Analysis and Standard Parameters may Increase (18)F-FET PET Accuracy Without Dynamic Scans. Eur Radiol (2017) 27:2916–27. doi: 10.1007/s00330-016-4638-2 27853813

[22] Liu Q Sun D Li N Kim J Feng D Huang G. Predicting EGFR Mutation Subtypes in Lung Adenocarcinoma Using (18)F-FDG PET/CT Radiomic Features. Transl Lung Cancer Res (2020) 9:549–62. doi: 10.21037/tlcr.2020.04.17 PMC7354146 32676319

[23] Antunovic L De Sanctis R Cozzi L Kirienko M Sagona A Torrisi R. PET/CT Radiomics in Breast Cancer: Promising Tool for Prediction of Pathological Response to Neoadjuvant Chemotherapy. Eur J Nucl Med Mol Imaging (2019) 46:1468–77. doi: 10.1007/s00259-019-04313-8 30915523

[24] Huang YQ Liang CH He L Tian J Liang CS Chen X. Development and Validation of a Radiomics Nomogram for Preoperative Prediction of Lymph Node Metastasis in Colorectal Cancer. J Clin Oncol (2016) 34:2157–64. doi: 10.1200/JCO.2015.65.9128 27138577

[25] Tobaly D Santinha J Sartoris R Dioguardi Burgio M Matos C Cros J. CT-Based Radiomics Analysis to Predict Malignancy in Patients With Intraductal Papillary Mucinous Neoplasm (IPMN) of the Pancreas. Cancers (Basel) (2020) 12:3089. doi: 10.3390/cancers12113089 PMC7690711 33114028

[26] Uhlig J Leha A Delonge LM Haack AM Shuch B Kim HS. Radiomic Features and Machine Learning for the Discrimination of Renal Tumor Histological Subtypes: A Pragmatic Study Using Clinical-Routine Computed Tomography. Cancers (Basel) (2020) 12:3010. doi: 10.3390/cancers12103010 PMC7603020 33081400

[27] Unterrainer M Vettermann F Brendel M Holzgreve A Lifschitz M Zahringer M. Towards Standardization of (18)F-FET PET Imaging: Do We Need a Consistent Method of Background Activity Assessment? EJNMMI Res (2017) 7:48. doi: 10.1186/s13550-017-0295-y 28560582 PMC5449315

[28] Pauleit D Floeth F Hamacher K Riemenschneider MJ Reifenberger G Muller HW. O-(2-[18F]Fluoroethyl)-L-Tyrosine PET Combined With MRI Improves the Diagnostic Assessment of Cerebral Gliomas. Brain (2005) 128:678–87. doi: 10.1093/brain/awh399 15689365

[29] van Griethuysen JJM Fedorov A Parmar C Hosny A Aucoin N Narayan V. Computational Radiomics System to Decode the Radiographic Phenotype. Cancer Res (2017) 77:e104–7. doi: 10.1158/0008-5472.CAN-17-0339 PMC5672828 29092951

[30] Zwanenburg A Leger S Valliéres M Löck S. Image Biomarker Standardisation Initiative. arXiv [Preprint] (2016). doi: 10.1016/S0167-8140(18)31291-X. arXiv:1612.07003.

[31] Seni G John IV. Ensemble Methods in Data Mining: Improving Accuracy Through Combining Predictions. Synthesis Lectures Data Min Knowledge Discovery (2010) 2:1–126. doi: 10.2200/S00240ED1V01Y200912DMK002

[32] Tibshirani R. The Lasso Method for Variable Selection in the COX Model. Stat Med (1997) 16:385–95. doi: 10.1002/(sici)1097-0258(19970228)16:4<385::aid-sim380>3.0.co;2-3 9044528

[33] Zhou ZR Wang WW Li Y Jin KR Wang XY Wang ZW. In-Depth Mining of Clinical Data: The Construction of Clinical Prediction Model With R. Ann Transl Med (2019) 7:796. doi: 10.21037/atm.2019.08.63 32042812 PMC6989986

[34] Li L Mu W Wang Y Liu Z Liu Z Wang Y. A Non-Invasive Radiomic Method Using (18)F-FDG PET Predicts Isocitrate Dehydrogenase Genotype and Prognosis in Patients With Glioma. Front Oncol (2019) 9:1183. doi: 10.3389/fonc.2019.01183 31803608 PMC6869373

[35] Bangalore Yogananda CG Shah BR Vejdani-Jahromi M Nalawade SS Murugesan GK Yu FF. A Novel Fully Automated MRI-Based Deep-Learning Method for Classification of IDH Mutation Status in Brain Gliomas. Neuro Oncol (2020) 22:402–11. doi: 10.1093/neuonc/noz199 PMC7442388 31637430

[36] Zhou W Zhou Z Wen J Xie F Zhu Y Zhang Z. A Nomogram Modeling (11)C-MET PET/CT and Clinical Features in Glioma Helps Predict IDH Mutation. Front Oncol (2020) 10:1200. doi: 10.3389/fonc.2020.01200 32850348 PMC7396495

[37] Wegmayr V Aitharaju S Buhmann J. Classification of Brain MRI With Big Data and Deep 3D Convolutional Neural Networks. Med Imaging: Computer-Aided Diagnosis (2018), 10575. doi: 10.1117/12.2293719

[38] Kratochwil C Combs SE Leotta K Afshar-Oromieh A Rieken S Debus J. Intra-Individual Comparison of (1)(8)F-FET and (1)(8)F-DOPA in PET Imaging of Recurrent Brain Tumors. Neuro Oncol (2014) 16:434–40. doi: 10.1093/neuonc/not199 PMC3922512 24305717

[39] Vettermann F Suchorska B Unterrainer M Nelwan D Forbrig R Ruf V. Non-Invasive Prediction of IDH-Wildtype Genotype in Gliomas Using Dynamic (18)F-FET PET. Eur J Nucl Med Mol Imaging (2019) 46:2581–9. doi: 10.1007/s00259-019-04477-3 31410540

[40] Kunz M Albert NL Unterrainer M la Fougere C Egensperger R Schuller U. Dynamic 18f-FET PET Is a Powerful Imaging Biomarker in Gadolinium-Negative Gliomas. Neuro Oncol (2019) 21:274–84. doi: 10.1093/neuonc/noy098 PMC6374762 29893965

[41] Haubold J Demircioglu A Gratz M Glas M Wrede K Sure U. Non-Invasive Tumor Decoding and Phenotyping of Cerebral Gliomas Utilizing Multiparametric (18)F-FET PET-MRI and MR Fingerprinting. Eur J Nucl Med Mol Imaging (2020) 47:1435–45. doi: 10.1007/s00259-019-04602-2 31811342

[42] Galldiks N Unterrainer M Judov N Stoffels G Rapp M Lohmann P. Photopenic Defects on O-(2-[18F]-Fluoroethyl)-L-Tyrosine PET: Clinical Relevance in Glioma Patients. Neuro Oncol (2019) 21:1331–8. doi: 10.1093/neuonc/noz083 PMC6784268 31077276

[43] Barresi V Eccher A Simbolo M Cappellini R Ricciardi GK Calabria F. Diffuse Gliomas in Patients Aged 55 Years or Over: A Suggestion for IDH Mutation Testing. Neuropathology (2020) 40:68–74. doi: 10.1007/s00259-019-04602-2 31758617

