# Supplementary material for: Integrated CT Radiomics Features Could Enhance the Efficacy of 18F-FET PET for Non-Invasive Isocitrate Dehydrogenase Genotype Prediction in Adult Untreated Gliomas: A Retrospective Cohort Study
Source: Front Oncol. 2021 Nov 19;11:772703. doi: 10.3389/fonc.2021.772703 (PMC8640504; doi:10.3389/fonc.2021.772703)
Supplement: Supplementary file 1 [file DataSheet_1.docx]

**Supplementary S1: Inclusion and exclusion criteria**

**Inclusion criteria**:1) positive ^18^F-FET PET imaging with satisfactory imaging quality; 2) histopathologically confirmed grade II-IV glioma according to 2016 WHO criteria; 3) availability of lesion *IDH* genotype results; 4) available preoperative structural MRI as the reference for manual delineation of the lesion area of interest; 5) no previous diagnostic or treatment procedures applied before the ^18^F-FET PET/CT investigation.

**Exclusion criteria**:1) juvenile patients; 2) intracranial lesions with histopathological diagnosis other than gliomas; 3) post-treatment glioma patients; 4) cases with hypo- or iso-metabolism of ^18^F-FET imaging compared to the brain background which is not applicable for the following tumor volume delineation procedures.

**Supplementary S2****: Report on image processing and radiomics features extraction**

| Acquisition and reconstruction | \| **Acquisition parameters** \| **Siemens Biograph 64** \| \| \| --- \| --- \| --- \| \| **PET** \| **CT** \| \| **^18^F-FET activity (MBq)** \| 370 ± 30 MBq \| – \| \| **Crystal** \| LSO \| – \| \| **Reconstruction** \| FBP \| – \| \| **Matrix (pixels)** \| 168×168 \| 512×512 \| \| **Resolution (mm)** \| 2.04 \| 0.59 \| \| **Slice thickness (mm)** \| 5 \| 5 \| \| **Slices** \| 148 \| 148 \| \| **Interslice gap (mm)** \| 1.5 \| 1.5 \| \| **Voltage (kV)** \| – \| 120 \| \| **Tube current (mA)** \| – \| 150 \| \| **Convolution kernel** \| – \| H30S \| |
| --- | --- | --- | --- | --- | --- | --- | --- | --- | --- | --- | --- | --- | --- | --- | --- | --- | --- | --- | --- | --- | --- | --- | --- | --- | --- | --- | --- | --- | --- | --- | --- | --- | --- | --- | --- | --- | --- | --- | --- |
| Approach | The images were analysed as a volume (3D). |
| Process structure | Image acquisition -> reconstruction ->anonymization -> segmentation -> export -> radiomics analysis -> feature calculation report |
| Software | PyRadiomics (https://github.com/Radiomics/pyradiomics) |
| Data availability | All the original patient DICOM files are stored in the institutional PACS. Anonymised DICOM files are stored on the Department hard disk. The calculations of the features are stored in the repository (link). |
| **Data conversion** | |
| Procedure | None |
| **Image post-acquisition processing** | |
| Procedure | None |
| **Segmentation** | |
| ROI | The VOI included the primary tumour lesion. Radiomics features were calculated on both PET and CT images within the same VOI. |
| Procedure | The ROIs were manually segmented using ITK-SNAP software(http://www.itksnap.org/pmwiki/pmwiki.php) |
| **Interpolation** |  |
| Voxel dimensions | PET: 2.5*2.5*2.5mm^3^;  CT: 1.5*1.5*1.5 mm^3^ |
| Image interpolation method | sitkBSpline |
| Image intensity rounding | The intensity of each image was normalised by its mean value with standard deviation to avoid individual heterogeneity. |
| ROI interpolation method | sitkBSpline |
| ROI partial volume | Not applicable |
| **Discretisation** |  |
| Discretisation method | CT: fixed binwidth with 0.5  PET: fixed binwidth with 0.05 |
| **Feature calculation** |  |
| Feature set | \| **Shape and Size Features(3D)** \| \| --- \| \| Elongation  Flatness  LeastAxisLength  MajorAxisLength  MinorAxisLength  Maximum2DDiameterColumn  Maximum2DDiameterRow  Maximum2DDiameterSlice  Maximum3DDiameter  MeshVolume  Sphericity  SurfaceArea  SurfaceVolumeRatio  VoxelVolume \| \| **First Order Features** \| \| firstorder_10Percentile  firstorder_90Percentile  firstorder_Energy  firstorder_Entropy  firstorder_InterquartileRange  firstorder_Kurtosis  firstorder_Maximum  firstorder_MeanAbsoluteDeviation  firstorder_Mean  firstorder_Median  firstorder_Minimum  firstorder_Range  firstorder_RobustMeanAbsoluteDeviation  firstorder_RootMeanSquared  firstorder_Skewness  firstorder_TotalEnergy  firstorder_Uniformity  firstorder_Variance \| \| **Texture Features** \| \| **Gray Level Co-occurrence Matrix (glcm)** \| \| glcm_Autocorrelation  glcm_JointAverage  glcm_ClusterProminence  glcm_ClusterShade  glcm_ClusterTendency  glcm_Contrast  glcm_Correlation  glcm_DifferenceAverage  glcm_DifferenceEntropy  glcm_DifferenceVariance  glcm_JointEnergy  glcm_JointEntropy  glcm_Imc1 (Informational Measure of Correlation 1)  glcm_Imc2 (Informational Measure of Correlation 2)  glcm_Idm (Inverse Difference Moment)  glcm_Idmn (Inverse Difference Moment Normalized)  glcm_Id (Inverse Difference)  glcm_Idn (Inverse Difference Normalized)  glcm_InverseVariance  glcm_MaximumProbability  glcm_SumEntropy  glcm_SumSquares \| \| **Grey-Level Run Length Matrix (glrlm)** \| \| glrlm_GrayLevelNonUniformity  glrlm_GrayLevelNonUniformityNormalized  glrlm_GrayLevelVariance  glrlm_HighGrayLevelRunEmphasis  glrlm_LongRunEmphasis  glrlm_LongRunHighGrayLevelEmphasis  glrlm_LongRunLowGrayLevelEmphasis  glrlm_LowGrayLevelRunEmphasis  glrlm_RunEntropy  glrlm_RunLengthNonUniformity  glrlm_RunLengthNonUniformityNormalized  glrlm_RunPercentage  glrlm_RunVariance  glrlm_ShortRunEmphasis  glrlm_ShortRunHighGrayLevelEmphasis  glrlm_ShortRunLowGrayLevelEmphasis \| \| **Gray Level Size Zone Matrix (glszm)** \| \| glszm_GrayLevelNonUniformity  glszm_GrayLevelNonUniformityNormalized  glszm_GrayLevelVariance  glszm_HighGrayLevelZoneEmphasis  glszm_LargeAreaEmphasis  glszm_LargeAreaHighGrayLevelEmphasis  glszm_LargeAreaLowGrayLevelEmphasis  glszm_LowGrayLevelZoneEmphasis  glszm_SizeZoneNonUniformity  glszm_SizeZoneNonUniformityNormalized  glszm_SmallAreaEmphasis  glszm_SmallAreaHighGrayLevelEmphasis  glszm_SmallAreaLowGrayLevelEmphasis  glszm_ZoneEntropy  glszm_ZonePercentage  glszm_ZoneVariance \| \| **Gray Level Dependence Matrix (gldm)** \| \| gldm_DependenceEntropy  gldm_DependenceNonUniformity  gldm_DependenceNonUniformityNormalized  gldm_DependenceVariance  gldm_GrayLevelNonUniformity  gldm_GrayLevelVariance  gldm_HighGrayLevelEmphasis  gldm_LargeDependenceEmphasis  gldm_LargeDependenceHighGrayLevelEmphasis  gldm_LargeDependenceLowGrayLevelEmphasis  gldm_LowGrayLevelEmphasis  gldm_SmallDependenceEmphasis  gldm_SmallDependenceHighGrayLevelEmphasis  gldm_SmallDependenceLowGrayLevelEmphasis \| \| **[Neighbouring Gray Tone Difference Matrix](" \l "radiomics.ngtdm.RadiomicsNGTDM" \o "radiomics.ngtdm.RadiomicsNGTDM) (ngtdm)** \| \| ngtdm_Busyness  ngtdm_Coarseness  ngtdm_Complexity  ngtdm_Contrast  ngtdm_Strength \| |
| Feature parameters | Texture matrices are built in 3D following the merging strategy (see IBSI reference document). |
| Standardization | Features values have been checked with the most up-to-date consensus of the IBSI benchmark values. |

**Supplementary S3: Detailed demographic and clinical data of all patients**

| **Number** | **Sex** | **Age** | **Location** | **Pathology** | **WHO grade** | **IDH1 status** | **Treatment** | **IDH1/2 sequencing** | **pTERT** | **MGMT** |
| --- | --- | --- | --- | --- | --- | --- | --- | --- | --- | --- |
| 1 | F | 41 | Frontal right | Diffuse astrocytoma | II | Wildtype | Total resection | Wildtype | Mutant | Unmethylated |
| 2 | M | 63 | Temporal right | Diffuse astrocytoma | II | Wildtype | Subtotal resection | Wildtype | Mutant | Unmethylated |
| 3 | M | 48 | Frontal right | Oligodendroglioma,NOS | II | R132H Mutation | Total resection | R132H Mutation | Mutant | Methylated |
| 4 | M | 30 | Brain stem | Astrocytoma | II | Wildtype | Sterotactic biopsy | N/A | N/A | N/A |
| 5 | M | 32 | Frontal right | Oligodendroglioma,NOS | II | R132H Mutation | Total resection | R132H Mutation | Mutant | Methylated |
| 6 | F | 35 | Frontal-insular left | Diffuse astrocytoma | II | Wildtype | Subtotal resection | N/A | N/A | N/A |
| 7 | F | 60 | Frontal left | Oligodendroglioma,NOS | II | R132H Mutation | Total resection | R132H Mutation | Mutant | Unmethylated |
| 8 | F | 49 | Temporal-thalamus left | Diffuse astrocytoma | II | Wildtype | Partial resection | N/A | N/A | N/A |
| 9 | F | 49 | Cerebellum left | Diffuse astrocytoma | II | Wildtype | Sterotactic biopsy | N/A | N/A | N/A |
| 10 | M | 56 | Frontal left | Oligodendroglioma,NOS | II | R132H Mutation | Subtotal resection | N/A | N/A | N/A |
| 11 | F | 29 | Frontal left | Diffuse astrocytoma | II | R132H Mutation | Total resection | R132H Mutation | Wildtype | Methylated |
| 12 | F | 35 | Frontal left | Oligodendroglioma,NOS | II | R132H Mutation | Total resection | N/A | N/A | N/A |
| 13 | M | 49 | Frontal left | Oligodendroglioma,NOS | II | R132H Mutation | Subtotal resection | N/A | N/A | N/A |
| 14 | F | 51 | Frontal left | Diffuse astrocytoma | II | Wildtype | Total resection | N/A | N/A | N/A |
| 15 | M | 23 | Temporal left | Diffuse astrocytoma | II | Wildtype | Total resection | N/A | N/A | N/A |
| 16 | F | 32 | Frontal-parietal right | Diffuse astrocytoma | II | Wildtype | Subtotal resection | N/A | N/A | N/A |
| 17 | M | 24 | Frontal left | Diffuse astrocytoma | II | Wildtype | Total resection | N/A | N/A | N/A |
| 18 | M | 52 | Frontal-parietal right | Oligodendroglioma,NOS | II | R132H Mutation | Subtotal resection | N/A | N/A | N/A |
| 19 | F | 29 | Frontal left | Oligodendroglioma,NOS | II | R132H Mutation | Total resection | R132H Mutation | Mutant | Methylated |
| 20 | F | 31 | Frontal left | Diffuse astrocytoma | II | R132H Mutation | Total resection | R132H Mutation | Mutant | Methylated |
| 21 | F | 51 | Frontal left | Oligodendroglioma,NOS | II | R132H Mutation | Total resection | R132H Mutation | Mutant | Methylated |
| 22 | M | 54 | Frontal right | Diffuse astrocytoma | II | Wildtype | Sterotactic biopsy | N/A | N/A | N/A |
| 23 | F | 45 | Parietal right | Diffuse astrocytoma | II | Wildtype | Sterotactic biopsy | Wildtype | Mutant | Methylated |
| 24 | M | 39 | Temporal right | Oligodendroglioma,NOS | II | R132H Mutation | Total resection | R132H Mutation | Mutant | Methylated |
| 25 | F | 44 | Temporal-insular right | Oligodendroglioma,NOS | II | R132H Mutation | Subtotal resection | R132H Mutation | Mutant | Methylated |
| 26 | M | 28 | Frontal left | Diffuse astrocytoma | II | R132H Mutation | Sterotactic biopsy | R132H Mutation | Wildtype | Methylated |
| 27 | M | 39 | Frontal right | Diffuse astrocytoma | II | R132H Mutation | Total resection | R132H Mutation | Mutant | Methylated |
| 28 | M | 42 | Frontal left | Oligodendroglioma,NOS | II | R132H Mutation | Total resection | R132H Mutation | Mutant | Methylated |
| 29 | M | 52 | Frontal-temporal-insular right | Oligodendroglioma,NOS | II | R132H Mutation | Subtotal resection | R132H Mutation | Mutant | Methylated |
| 30 | M | 24 | Frontal-temporal left | Diffuse astrocytoma | II | R132H Mutation | Total resection | R132H Mutation | Wildtype | Unmethylated |
| 31 | F | 45 | Frontal left | Diffuse astrocytoma | II | Wildtype | Sterotactic biopsy | Wildtype | Mutant | Unmethylated |
| 32 | F | 66 | Frontal-temporal left | Anaplastic astrocytoma | III | Wildtype | Total resection | Wildtype | Mutant | Methylated |
| 33 | M | 29 | Frontal-temporal-insular left | Anaplastic astrocytoma | III | Wildtype | Partial resection | Wildtype | Mutant | Unmethylated |
| 34 | M | 66 | Brain stem | Anaplastic astrocytoma | III | Wildtype | Sterotactic biopsy | N/A | N/A | N/A |
| 35 | M | 63 | Frontal-insular right | Anaplastic astrocytoma | III | Wildtype | Subtotal resection | Wildtype | Mutant | Unmethylated |
| 36 | M | 52 | Frontal left | Anaplastic astrocytoma | III | Wildtype | Total resection | N/A | N/A | N/A |
| 37 | M | 38 | Corpus callosum | Anaplastic astrocytoma | III | Wildtype | Sterotactic biopsy | Wildtype | Wildtype | Methylated |
| 38 | M | 55 | Frontal bilateral and corpus callosum | Anaplastic astrocytoma | III | R132H Mutation | Sterotactic biopsy | N/A | N/A | N/A |
| 39 | F | 67 | Temporal-insular left | Anaplastic astrocytoma | III | Wildtype | Total resection | N/A | N/A | N/A |
| 40 | M | 31 | Frontal-parietal right | Diffuse astrocytoma | III | Wildtype | Sterotactic biopsy | N/A | N/A | N/A |
| 41 | F | 20 | Basal ganglia-thalamus left | Anaplastic astrocytoma | III | Wildtype | Sterotactic biopsy | Wildtype | Wildtype | Methylated |
| 42 | F | 30 | Occipital-thalamus bilateral and corpus callosum | Anaplastic astrocytoma | III | Wildtype | Sterotactic biopsy | N/A | N/A | N/A |
| 43 | M | 68 | Frontal right | Anaplastic astrocytoma | III | R132H Mutation | Total resection | R132H Mutation | Wildtype | Methylated |
| 44 | M | 62 | Basal ganglia-thalamus right | Anaplastic astrocytoma | III | Wildtype | Sterotactic biopsy | Wildtype | Mutant | Unmethylated |
| 45 | F | 24 | Frontal left | Anaplastic astrocytoma | III | Wildtype | Sterotactic biopsy | N/A | N/A | N/A |
| 46 | M | 17 | Cerebellum right | Glioblastoma | IV | Wildtype | Partial resection | N/A | N/A | N/A |
| 47 | F | 70 | Frontal left | Glioblastoma | IV | Wildtype | Total resection | N/A | N/A | N/A |
| 48 | M | 46 | Frontal-temporal-insular right | Glioblastoma | IV | Wildtype | Total resection | N/A | N/A | N/A |
| 49 | M | 29 | Thalamus right | Diffuse midline glioma | IV | Wildtype | Sterotactic biopsy | N/A | N/A | N/A |
| 50 | F | 21 | Thalamus right and brain stem | Diffuse midline glioma | IV | Wildtype | Sterotactic biopsy | N/A | N/A | N/A |
| 51 | M | 30 | Thalamus left | Glioblastoma | IV | Wildtype | Sterotactic biopsy | Wildtype | Mutant | Unmethylated |
| 52 | M | 57 | Basal ganglia-thalamus left | Glioblastoma | IV | Wildtype | Subtotal resection | N/A | N/A | N/A |
| 53 | M | 51 | Temporal bilateral and corpus callosum | Glioblastoma | IV | Wildtype | Sterotactic biopsy | N/A | N/A | N/A |
| 54 | M | 19 | Frontal-insular right | Glioblastoma | IV | Wildtype | Total resection | Wildtype | Wildtype | Methylated |
| 55 | M | 61 | Frontal right | Glioblastoma | IV | Wildtype | Subtotal resection | Wildtype | Mutant | Unmethylated |
| 56 | M | 29 | Frontal left | Glioblastoma | IV | Wildtype | Total resection | Wildtype | Wildtype | Unmethylated |
| 57 | M | 18 | Frontal right and corpus callosum | Glioblastoma | IV | Wildtype | Sterotactic biopsy | Wildtype | Wildtype | Methylated |
| 58 | M | 27 | Temporal right | Glioblastoma | IV | Wildtype | Partial resection | Wildtype | Wildtype | Unmethylated |

Abbreviations: *IDH*, isocitrate dehydrogenase; p*TERT*, telomerase reverse transcriptase promoter; *MGMT,* O^6^-methylguanine-DNA methyltransferase; mut, mutant; wt, wild-type; M, male; F, female; N/A, not applicable.
